# Therapeutic Sequences in the Treatment of High-Risk Prostate Cancer: Paving the Way Towards Multimodal Tailored Approaches

**DOI:** 10.3389/fonc.2021.732766

**Published:** 2021-08-04

**Authors:** Giulia Marvaso, Giulia Corrao, Mattia Zaffaroni, Matteo Pepa, Matteo Augugliaro, Stefania Volpe, Gennaro Musi, Stefano Luzzago, Francesco Alessandro Mistretta, Elena Verri, Maria Cossu Rocca, Matteo Ferro, Giuseppe Petralia, Franco Nolè, Ottavio De Cobelli, Roberto Orecchia, Barbara Alicja Jereczek-Fossa

**Affiliations:** ^1^Division of Radiation Oncology, IEO, European Institute of Oncology IRCCS, Milan, Italy; ^2^Department of Oncology and Hemato-Oncology, University of Milan, Milan, Italy; ^3^Department of Urology, IEO, European Institute of Oncology IRCCS, Milan, Italy; ^4^Department of Medical Oncology, IEO, European Institute of Oncology IRCCS, Milan, Italy; ^5^Precision Imaging and Research Unit, Department of Medical Imaging and Radiation Sciences, IEO, European Institute of Oncology IRCCS, Milan, Italy; ^6^Medical Oncology Division of Urogenital & Head & Neck Tumors, IEO, European Institute of Oncology IRCCS, Milan, Italy; ^7^Scientific Directorate, IEO, European Institute of Oncology IRCCS, Milan, Italy

**Keywords:** high risk prostate cancer, personalized medicine, hadrontherapy, narrative review, second-generation antiandrogens, iPARP treatment

## Abstract

Various definitions are currently in use to describe high-risk prostate cancer. This variety in definitions is important for patient counseling, since predicted outcomes depend on which classification is applied to identify patient’s prostate cancer risk category. Historically, strategies for the treatment of localized high-risk prostate cancer comprise local approaches such as surgery and radiotherapy, as well as systemic approaches such as hormonal therapy. Nevertheless, since high-risk prostate cancer patients remain the group with higher-risk of treatment failure and mortality rates, nowadays, novel treatment strategies, comprising hypofractionated-radiotherapy, second-generation antiandrogens, and hadrontherapy, are being explored in order to improve their long-term oncological outcomes. This narrative review aims to report the current management of high-risk prostate cancer and to explore the future perspectives in this clinical setting.

## Introduction

**Figure 1:**
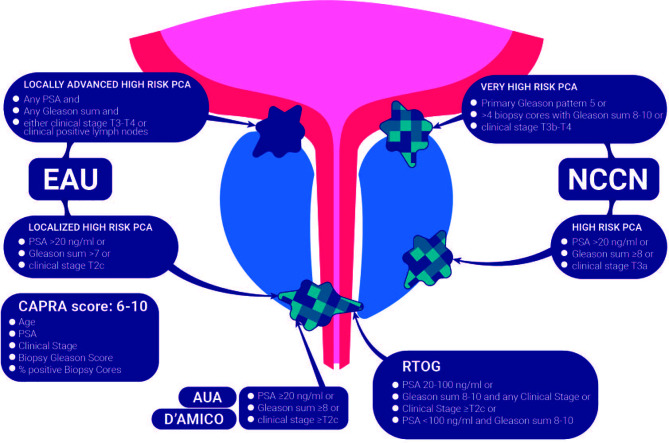
Summary of the currently available classifications for high-risk prostate cancer.

High-risk prostate cancer (HR PCa) is defined according to the pathological grade of the disease (Gleason score (GS)), prostate specific antigen (PSA) value, and disease extent (1). A summary of the currently available classifications is reported in **Figure 1**. The heterogeneity of patients included in this risk class accounts for the variety of expected outcomes with widely reported percentages of biochemical and metastatic recurrences (2–4). This non-homogeneity in definitions is important for patient counseling, as reported outcomes depend on which classification is applied to identify patient PCa risk category (5). Consequently, the ideal management strategy is presently unclear but is likely to involve a multimodal approach. Given that HR PCa is associated with early treatment failure and metastatic relapse of disease after definitive therapies, with low overall survival (OS) rates (6), novel treatment strategies are being explored in order to improve their long-term oncological outcomes.

This narrative review aims to investigate the evolution of the management of HR PCa. In particular, the first section reports some of the old, and partially solved, questions: (i) radiotherapy (RT) *vs* surgery, (ii) appropriate androgen deprivation therapy (ADT) duration, and (iii) prophylactic pelvic irradiation. The subsequent section focuses on future perspectives and novel treatment strategies including (i) ultra-hypofractionated RT, (ii) second-generation antiandrogens and poly (ADP-ribose) polymerase inhibitors (iPARP), and (iii) particle therapy.

## Old Scenarios

### Local Approaches: RT *vs* Surgery

It is well established that treatment options for localized HR PCa should include a definitive local strategy, with 87 and 57% cancer-specific survival (CSS) rates observed among treated and untreated patients, respectively (7). In accordance to these data, both National Comprehensive Cancer Network (NCCN) (8) and European Association of Urology (EAU) guidelines (1) strongly recommend a definitive treatment, stratifying patients in accordance with their life expectancy (with a threshold of 5 and 10 years, respectively). Guidelines’ recommendations include radical prostatectomy (RP) + pelvic lymph node dissection (PLDN) or external beam RT (EBRT) + long-term ADT (1.5–3 years) ± a brachytherapy boost (8).

Since evidence from randomized controlled trials (RCTs) comparing surgery and EBRT still lacks, nowadays no consensus exists on the best treatment choice. A recent international multidisciplinary systematic review (9) was unable to demonstrate the superiority of such approaches as primary local therapy. The ongoing randomized phase III SPCG-15 trial (10) comparing CSS of locally advanced PCa patients treated with RP + ePLDN ± EBRT or EBRT + ADT is expected to provide evidence on this aspect.

### EBRT + ADT

Androgen suppression is an established strategy for the treatment of HR PCa. Usually it is accomplished *via* the use of luteinizing hormone–releasing hormone (LHRH) analogs or antagonists, ± antiandrogens.

It is widely recognized that improving OS may be obtained by adding ADT to RT in HR PCa patients with a life expectancy >10 years (11–13). The latter evidence is supported by an RCT showing 10-year OS of 40 to 58% among patients receiving RT alone or combined treatments, respectively (p = 0.0004).

However, the appropriate ADT duration is actually undefined, considering in particular its relation with the patient’s reported quality of life (QoL). Two studies (14, 15) addressing this issue have reported that long-term ADT (18–36 months) has better oncological outcomes with respect to short-term ADT. Conversely, a recent phase III RCT (16) comparing long- (36 months) and intermediate- (18 months) term ADT did not observe significant difference in clinical outcomes (CSS and distant metastases development), but only a benefit in QoL for intermediate group. Currently, age, performance status, comorbidities, and the number of poor prognostic factors are recommended to be considered for establishing the ADT duration in clinical practice. In general, the current evidence supports the fact that any ADT duration is better than no ADT at all (12, 17–19), that long-term ADT (e.g., 3 years) is slightly better in OS than a short duration (6 months) (15), but it remains debated whether a duration of <3 years (16) in some patients or >3 years in very HR patients is more appropriate.

### Whole-Pelvis Irradiation

The efficacy of dose-escalated EBRT to the prostate alone in patients with HR disease might be limited by the increased likelihood of occult pelvic lymph node metastases outside of the radiation field.

In the past years, different studies showed opposing evidences on the administration of prophylactic whole-pelvis RT, such as the study by Roach et al. (20), which reported a benefit for neoadjuvant ADT followed by WPRT in contrast with Lawton and Colleagues (21). who failed to demonstrate a benefit in men without positive lymph nodes.

As a result, there is no consensus on WPRT administration. Ideally, WPRT should erase micrometastatic lesions, improving locoregional control (LRC) and OS.

However, the phase III POP-RT trial (22), including 224 patients affected by HR-PCa receiving hypofractionated RT to the prostate and randomized to include or exclude the whole pelvis, confirms the role of prophylactic WPRT in improving the biochemical failure-free survival and disease-free survival without a significant reported benefit in OS rates.

For this purpose, two RCTs (23, 24) are ongoing and might provide more robust evidence on the issue. In particular, with an expected trial end date of August 2021, PIVOTAL-boost is a multicenter four-arm superiority phase III trial for intermediate and HR PCa patients with failure-free survival as primary endpoint through administration of intensity-modulated RT (IMRT) on prostate ± pelvic and prostate boost on dominant lesion(s). Similarly, the RTOG 0924, a phase III randomized trial, with primary outcome measure stated as OS assigning unfavorable intermediate or favorable HR PCa patients to ADT + EBRT ± WPRT. The estimated primary completion date is July 2027.

Waiting for results from these RCTs, radiation oncologists are divided on the best strategy in the clinical practice.

In the era of tailored treatments, in order to avoid unnecessarily larger treatment fields, Gallium 68 prostate specific membrane antigen (Ga^68^ PSMA-PET) and whole-body Magnetic Resonance Imaging (MRI) could help to early identify pelvic lymph node localizations if PSA is still detectable (25, 26).

Such image-guidance techniques, mapping microscopic disease with improved sensitivity and sensibility, could also allow for dose escalation to nodes outside the conventional volumes (27).

## New Scenarios

### Hypofractionated and Ultra-Hypofractionated RT

Based on the radiosensibility of the PCa cells, it has been largely demonstrated that hypofractionation and extreme hypofractionation are safe and effective in low and intermediate risk PCa (28–31). In fact, the strong biologic rationale behind hypofractionation is based on the theory that the slow proliferation of PCa cells results in a different radiation response compared to other human cancers (32, 33). Therefore, the inability of PCa cells to overcome the higher rate of DNA damage induced by each fraction translates into an increased sensitivity to higher doses per fraction.

Up to date, multiple clinical trials have shown the effectiveness and the safety of moderate/standard hypofractionation for PCa treatment both in terms of oncological outcomes and toxicity (28–30, 34–36). Currently, thanks to the advent of modern techniques such as IMRT, highly conformal doses can be delivered to the target without affecting normal tissues, tilting the risk/benefit ratio more favorably towards RT (37, 38). Based on results from CHHiP and HYPRO (30) trials, hypofractionated schemes represent a valid treatment option for HR PCa. The number of studies involving extreme hypofractionation (defined as the delivery of 5–10 Gy/fraction in four to seven fractions) is relatively low, and a direct comparison of different hypofractionation schemes is still lacking. Therefore, despite being cited in clinical practice guidelines next to moderate hypofractionation schemes, the current level of evidence is too low to implement extreme hypofractionation as a standard of care (39).

Overall, while hypofractionation is a well-established practice, the comparison and reproducibility of published studies regarding ultra-hypofractionation in HR PCa are of difficult interpretation due to the many limitations that must be taken into account in order to draw reliable conclusions including (i) the small number of prospective studies, (ii) the paucity and quality of the reported data, (iii) the lack of technical RT delivery data analyses, (iv) the scarcity of QoL data, and (v) the not consistent definition of HR PCa throughout the studies.

To explore the feasibility of ultra-hypofractionated regimens, the Hypo-RT trial (40) enrolled 1,200 intermediate and HR PCa with about 10% being HR patients who randomly received ultra-hypofractionated (42.7 Gy in seven fractions) or conventional RT (78 Gy in 39 fractions). Results at 5 years reported a failure-free survival rate of 84% in both arms (no ADT was allowed). *Post-hoc* subgroup analyses failed to show significant interactions between risk and treatment group.

One hundred intermediate or HR PCa patients enrolled in the hypo-FLAME (41) prospective phase II trial received 35 Gy in five weekly fractions to the whole prostate gland with an integrated boost up to 50 Gy to the dominant intraprostatic lesion(s). In the study, no grade (G) 3 acute genitourinary (GU) or gastrointestinal (GI) toxicities were observed.

One of the main issues about ultra-hypofractionated regimens in HR PCa regards the expected toxicity following prophylactic WPRT.

In this scenario, the FASTR and FASTR-2 trials (42, 43) aimed to evaluate acute toxicity after ultra-hypofractionated RT.

In the first FASTR study, 15 men matched the inclusion criteria. RT was delivered to the prostate gland (40 Gy) and simultaneously to pelvic nodes with a dose of 25 Gy. Nine patients experienced grade (G) ≥2 gastrointestinal (GI) or genitourinary (GU) toxicities and five men reported G≥2 GI and GU toxicity at 6 months.

In the FASTR-2, a lower dose was given on prostate gland (35 Gy), no WPRT was included, and a smaller posterior planning target volume (PTV) (4 mm *vs* 5 mm) margin was provided. As expected, the FASTR-2 showed lower grades of GI/GU toxicities with respect to the FASTR trial. One patient reported a G2 GI acute toxicity, and no cases of G2 GI late toxicities were counted. Nine and five patients reported acute and late G2 GU toxicities, respectively.

From the reported results, ultra-hypofractionated RT schemes on prostate gland seems a feasible, safe, and effective treatment options for HR PCa patients.

### New Potential Drugs

In order to provide novel and personalized treatment strategies and to improve QoL and long-term outcomes, drugs currently administered in metastatic settings are in study for localized and locally advanced HR PCa patients. In particular, based on the striking results in advanced PCa (44, 45), both second-generation ADT and poly (adenosine diphosphate-ribose) polymerase 1 inhibitors (iPARP) are currently considered as potential candidates to be administered in early stages of HR PCa in order to improve oncological outcomes in this controversial setting.

#### Second-Generation ADT

First-generation antiandrogens established androgen receptor (AR) blockade as a therapeutic strategy but do not completely abrogate its activity (46). Despite the immediate palliative effect achieved with ADT, patients tend to relapse within a few years due to alternative mechanisms of AR signaling, AR amplification/alternative splicing, intratumoral androgen production, or adrenal gland testosterone production.

Nowadays, efficacy and potency have been improved by the development of second-generation antiandrogen therapies which exhibits (i) increased specificity to the AR over other steroidal receptors, (ii) higher affinity than the first generation, (iii) exclusively antagonistic activity towards the AR, and in turn, (iv) no androgen withdrawal syndrome. These second-generations molecules include androgen biosynthesis inhibitor abiraterone acetate and direct AR blockers, such as enzalutamide, apalutamide, and darolutamide, blocking AR with 6–9-fold greater affinity than the first-generation agents (47, 48). Hypothetically, the association of second-generation androgen receptor pathway inhibitors with EBRT can result in an added benefit for patients, especially those at a high risk of micrometastatic disease. From this perspective, abiraterone [STAMPEDE (49)], enzalutamide (ENZARAD), and apalutamide (ATLAS (50), ARNEO (51), PROTEUS) are currently under investigation to treat HR PCa, in combination with local approaches (**Table 1**).

**Table 1:** Ongoing trials.

Clinical trial ID	Description	Intervention	Size	Status	Primary outcome
SECOND GENERATION ADT					
ARNEO trial (NCT03080116)	Interventional, single center, phase II, randomized, double-blind, placebo controlled	degarelix + apalutamide *vs* degarelix + placebo	84 (estimated)	Recruiting	Minimal residual disease after 12 weeks of neoadjuvant therapy
ATLAS trial (NCT02531516)	Interventional, multicenter, phase III, randomized, double-blind, placebo-controlled	Apalutamide + placebo + RT *vs* placebo + ADT + RT	1,503 (actual)	Not recruiting	Metastasis-free survival
ENZARAD trial (NCT02446444)	Interventional, phase III, randomized, open label	Enzalutamide + LHRHa + RT *vs* conventional NSAA + LHRHa + RT	802 (actual)	Not recruiting	Metastasis-free survival
PROTEUS trial (NCT03767244)	Interventional, phase III, randomized, double-blind, placebo controlled	Apalutamide + ADT + RP + pLND *vs* placebo + ADT + RP + pLND	1,500 (estimated)	Recruiting	Pathologic complete response (pCR) and metastasis-free survival
iPARP					
NADIR trial (NCT04037254)	Interventional, phase II, randomized, open label	ADT + IMRT *vs* niraparib + ADT + IMRT	180 (estimated)	Not recruiting	Maintenance of disease-free state
PARTICLE THERAPY					
NCT02672449	Prospective, multicenter, phase II, open label	Carbon ion boost followed by photon RT	65 (estimated)	Recruiting	G3 or G4 adverse events according to the RTOG / EORTC scale

ADT, androgen deprivation therapy; G, grade; IMRT, intensity-modulated RT; LHRHa, luteinizing hormone–releasing hormone analog; NSAA, non-steroidal anti-androgen; pCR, pathologic complete response; pLND, pelvic lymph node dissection; RP, radical prostatectomy; RT, radiotherapy.

In general, since the potential for the novel antiandrogens as standalone therapeutic had reached a plateau for use in advanced PCa, it is far more likely that the next wave of therapeutic investigation will be focused on the combination of this class of antiandrogen therapy with other treatments such as RT and chemotherapy. In fact, as reported by Elsesy et al. (52), the use of second-generation antiandrogens radiosensitizes PCa *via* the inhibition of the DNA double-strand break repair machinery. These results are in accordance with recent preclinical studies (53) reporting that enzalutamide has a radiosentization role, increasing the effect of ionizing radiation.

To foster this evidence, Zhang and Colleagues (54) demonstrated that apalutamide acts as a radiosensitizer in both androgen-dependent PCa and castration-resistant PCa models. These results suggest that apalutamide can be used in combination with EBRT for the treatment of androgen-dependent localized PCa (50).

#### PARP Inhibitors

One of the potential reasons for radioresistance is the ability of tumor cells to repair the damage inflicted by radiotherapy. Following the induction of DNA double-strand breaks (DSB) by ionizing radiation, cancer cells mount a rapid response involving an extensive network of pathways. This response involves the cellular machinery required to repair damaged DNA and allows the malignant cell to survive and retain its reproductive integrity. This network is broadly referred to as the DNA damage response (DDR). It is well-known how high rates of genomic mutations in DDR genes result directly related to multiple malignancies (55–57), and more recently, it has been suggested that tumors with such homologous recombination defects may be sensitive to iPARP (58, 59).

Currently, there are multiple agents such as olaparib, niraparib, and rucaparib (58, 60, 61) that target the DDR pathway. Among these iPARP, olaparib and rucaparib have been shown to be effective in men with metastatic castration-resistant PCa (mCRPC) (44, 62, 63). Since DDR pathway alterations were seen at similar rate between localized and metastatic PCa, it has been speculated that iPARP may also have a therapeutic effect in localized PCa (64).

To support this hypothesis, a 2019 study by Kim et al. (64) analyzed the DDR pathway alterations in localized PCa using The Cancer Genome Atlas (TCGA) public database. Their results highlighted that DDR alteration rate was surprisingly higher than suggested by previous studies (65, 66) and was associated with shorter OS in men with postoperatively HR features.

Some of the ongoing trials regrading iPARPs in localized PCa are reported in **Table 1**.

Overall, the above reported findings suggest that a dysregulated DDR pathway may occur earlier during PCa progression than previously thought and that available inhibitors of DDR pathway, such as iPARPs, may have an effective therapeutic role in localized PCa.

### Particle Therapy

Particle therapy has been gaining growing interest due to the particular physical and radiobiological properties of protons and other heavy ions, including carbon ions, compared to photons (67). Particularly, hadrontherapy with protons and carbon ions has been considered a suitable strategy for the treatment of localized and locally advanced PCa to reach high doses while maintaining a lower toxicity rate.

#### Carbon Ion Therapy

Carbon ion RT (CIRT) may represent an ideal treatment method for PCa due to the unique physical and biological advantages of carbon ion beams. The dose distribution of CIRT is most advantageous for EBRT techniques because of its superior dose characteristics (68). Firstly, steep dose gradients result in a better sparing of organs at risk (OARs) close to the target. Moreover, carbon ion beams have a high relative biological effectiveness (RBE), resulting from a high linear energy transfer, with their effect estimated to be approximately three times those of photons and protons (69, 70). Finally, carbon ions might affect radioresistant clusters and make them more sensitive to a subsequent photon therapy.

The first clinical trial of CIRT for PCa was initiated at the National Institute of Radiological Sciences (NIRS) in 1994, and the efficacy and feasibility of CIRT for localized PCa have been demonstrated through three phase I/II and two phase II clinical trials (71) at NIRS. The studies published from the Japanese centers represent an important starting point for the clinical use of carbon ions in this setting of patients (72, 73).

A 2017 study by Kasuya and Colleagues (74) analyzed the treatment outcomes of HR localized PCa treated with CIRT + ADT compared with standard treatment modalities, focusing on PCa‐specific mortality (PCSM). Despite differences in PCSM among the high‐risk groups, CIRT combined with ADT yielded relatively favorable treatment outcomes.

The first prospective observational study conducted at a facility other than NIRS is the study by Kawamura et al. (75), which reported low GU and GI toxicities with good biochemical control within 5 years following moderately hypofractionated CIRT for localized PCa.

The NCT02672449 is a prospective, multicentric, phase II open-label trial that might provide novel insights on a new mixed beam RT scheme of a carbon ion boost followed by pelvic photon RT (76), and details about this ongoing trial are reported in **Table 1**. Overall, data about CIRT in HR setting seems encouraging and could provide novel insight for the treatment of these patients.

#### Proton Therapy

Owing to the well-known unique dose distribution of protons (77), efforts have been made to adapt their benefits in PCa therapies. In particular, their ability to reduce irradiation to the adjacent OARs, thanks to the Bragg Peak (78–80), allows for a highly localized deposition of energy on the tumor (81).

As of today, two studies report data about PBRT in an HR setting.

Takagi et al. (82) reported the largest PBRT (± ADT) series in localized PCa with a 10-year follow-up. Among a cohort of 2,021 patients, a total of 792 belonged to HR or very HR groups. The control of PBRT resulted favorable, with a biochemical control rate of 68 and 62% in HR and very HR patients, respectively. Five-year OS was 96% in the HR group and 92% in the very HR cohort. The results of the study encourage the planning of novel prospective clinical trials.

In a smallest series, Arimura et al. (83) conducted a prospective cohort study on 218 patients with intermediate-risk and HR PCa declining ADT, receiving PBRT. Unexpectedly, results were similar to those of previous reported ones from studies concerning PBRT + ADT where in a PBRT setting, ADT for 12 months and 21 months was shown as preferable for HR PCa patients (84). Therefore, monotherapy PBRT can be considered as an optional treatment in this setting, even if studies that include more patients and longer follow-up are needed to clarify the definitive role of PBRT in the treatment of HR localized PCa.

In particular, there is an urgent need for randomized data comparing photon- *versus* proton-based EBRT head to-head for localized and HR PCa cancer to rigorously inform the debate surrounding proton therapy for PCa.

## Conclusions

Some of the old questions in the treatment of HR localized PCa seem to have been solved; nevertheless, modern treatment strategies bring with them novel questions that need to be addressed.

Multidisciplinary teams of urologists, medical oncologists, radiation oncologists, radiologists, and pathologists will be instrumental in shifting the treatment tide for the patients.

Integrative multimodal personalized treatment approaches inclusive of surgery, ultra-hypofractionated RT, hadrontherapy, and systemic therapy represent a valid potential way to improve long-term outcomes in patients with HR PCa.

## Author Contributions

GMa, GC, and MZ were responsible for conception and design of the study and wrote the first draft of the manuscript. SV and MP were responsible for data acquisition and wrote sections of the manuscript. MA wrote sections of the manuscript. All authors contributed to the article and approved the submitted version.

## Funding

Università degli Studi di Milano. This study was supported by a research grant from the Associazione Italiana per la Ricerca sul Cancro (AIRC) entitled“ Radioablation± hormonotherapy for prostate cancer oligorecurrences (RADIOSA trial): potential of imaging and biology” registered at ClinicalTrials.gov NCT03940235, approved by the Ethics Committee of IRCCS Istituto Europeo di Oncologia and Centro Cardiologico Monzino (IEO-997).

## Conflict of Interest

The authors declare that the research was conducted in the absence of any commercial or financial relationships that could be construed as a potential conflict of interest.

The handling editor declared a past co-authorship with one of the authors BJ.

## Publisher’s Note

All claims expressed in this article are solely those of the authors and do not necessarily represent those of their affiliated organizations, or those of the publisher, the editors and the reviewers. Any product that may be evaluated in this article, or claim that may be made by its manufacturer, is not guaranteed or endorsed by the publisher.

## Abbreviations

ADT, Androgen Deprivation Therapy; AR, Androgen Receptor; CIRT, Carbon Ion Radiotherapy; CSS, Cancer Specific Survival; DDR, DNA Damage Repair; EAU, European Association of Urology; EANM, European Association of Nuclear Medicine; EBRT, External Beam Radiotherapy; ePLND, Extended Pelvic Lymph Nodes Dissection; ESTRO, European Society for Therapeutic Radiation and Oncology; ESUR, European Society of Urogenital Radiology; G, Grade; GI, Gastrointestinal; GU, Genitourinary; GS, Gleason Score; HR PCa, High-risk Prostate Cancer; iPARP, Enzyme Poly ADP Ribose Polymerase inhibitors; IMRT, Intensity Modulated Radiotherapy; LCR, Local Control Rate; mCRPC, metastatic Castration-Resistant Prostate Cancer; NIRS, National Institute of Radiological Sciences; NCCN, National Comprehensive Cancer Network; OARs, Organ at Risk; OS, Overall Survival; PBRT, Proton Beam Radiotherapy; PCSM, Prostate Cancer Specific Mortality; PSA, Prostate Specific Antigen; PTV, Planning Target Volume; QoL, Quality of Life; RBE, Relative Biological Effectiveness; RCT(s), Randomized Clinical Trial(s); RP, Radical Prostatectomy; SBRT, Stereotactic Body Radiation Therapy; SIOG, International Society of Geriatric Oncology; TCGA, The Cancer Genome Atlas; WPRT, Whole Pelvis Radiotherapy.
